# Animal Interactions and the Emergence of Territoriality

**DOI:** 10.1371/journal.pcbi.1002008

**Published:** 2011-03-10

**Authors:** Luca Giuggioli, Jonathan R. Potts, Stephen Harris

**Affiliations:** 1 Bristol Centre for Complexity Sciences, University of Bristol, Bristol, United Kingdom; 2 Department of Engineering Mathematics, University of Bristol, Bristol, United Kingdom; 3 School of Biological Sciences, University of Bristol, Bristol, United Kingdom; New York University, United States of America

## Abstract

Inferring the role of interactions in territorial animals relies upon accurate recordings of the behaviour of neighbouring individuals. Such accurate recordings are rarely available from field studies. As a result, quantification of the interaction mechanisms has often relied upon theoretical approaches, which hitherto have been limited to comparisons of *macroscopic* population-level predictions from un-tested interaction models. Here we present a quantitative framework that possesses a *microscopic* testable hypothesis on the mechanism of conspecific avoidance mediated by olfactory signals in the form of scent marks. We find that the key parameters controlling territoriality are two: the average territory size, i.e. the inverse of the population density, and the time span during which animal scent marks remain active. Since permanent monitoring of a territorial border is not possible, scent marks need to function in the temporary absence of the resident. As chemical signals carried by the scent only last a finite amount of time, each animal needs to revisit territorial boundaries frequently and refresh its own scent marks in order to deter possible intruders. The size of the territory an animal can maintain is thus proportional to the time necessary for an animal to move between its own territorial boundaries. By using an agent-based model to take into account the possible spatio-temporal movement trajectories of individual animals, we show that the emerging territories are the result of a form of collective animal movement where, different to shoaling, flocking or herding, interactions are highly heterogeneous in space and time. The applicability of our hypothesis has been tested with a prototypical territorial animal, the red fox (*Vulpes vulpes*).

## Author Summary

How animals succeed in sharing and occupying space in an efficient way has always fascinated biologists. When occupying space involves marking and defending a given area, the animal is said to be territorial. By scent marking the locations that an animal visits, it conveys to a potential intruder that the area is claimed by another animal. Once an intruder encounters a foreign scent, it typically retreats from it to avoid an aggressive response by the resident animal. This is the so-called mechanism of conspecific avoidance. By considering this mechanism and the movement of the individual animals, we predict how territorial patterns are formed and maintained. Data and information on the red fox has served as a benchmark to test our predictions and has provided the experimental support to our theory. The implications of our results reach far beyond behavioural ecology, encompassing fields from epidemiology and conservation biology to social and state boundary dynamics in human society and ‘divide and conquer’ approaches to collective robotics.

## Introduction

Animal territoriality aims at excluding conspecifics from certain areas through the use of auditory, visual or olfactory signals as well as aggressive interactions [1]. Its widespread occurence across so many different taxa has prompted the question as to whether general mechanisms for such behaviour exist [2], [3]. Answering this question however has proved elusive, partly because territorial behaviour spans organizational levels from individual animals [4] to populations [5] and the ecosystem [6], but also because it requires an understanding of how conspecific avoidance processes observed at small spatial scales and short time scales generate extended and lasting territorial patterns. Such multi-scale dynamics are ubiquitous in ecology [7] and their explanation often requires a *microscopic* level description of the processes at play. Here we provide such an approach by building a stochastic individual-based model (probabilistic cellular automata [8]) that reveals how territorial boundaries are formed, and change position, from the animal movements and interactions mediated through olfactory signals (scent marks).

Although the importance of animal interactions in determining the shape and size of territories is now recognized [3], none of the recent studies have attempted [9]–[11] or succeeded in quantifying [12] *microscopically* how territories emerge from the movement and interaction between animals. These recent analyses in fact share the common feature of being *macroscopic* representations of the interaction processes since they do not account for the discreteness of the animal population and the interaction events [13], which occur locally and over short time periods. Key to our approach is in fact the recognition that conspecific avoidance, mediated through the deposition of olfactory signals, makes animal territories undergo the so-called *exclusion processes* [14], [15] whose dynamics demand an individual level description.

Here we explore the transient dynamics in the formation of animal territories, moving away from traditional approaches where territories are assumed to settle to a steady state [9]–[12] and moving towards a mechanistic explanation derived from the individual animal's social interactions. By keeping track of the locations where each animal wanders over the terrain, we show how scent serves as a short-term cue, explaining why territorial mammals regularly renew and refresh their scent marks. We also show how to apply our findings by using data from a well-studied territorial species, the red fox (*Vulpes vulpes*), and extract information about the persistence of scent marks.

## Results

**Figure 1:**
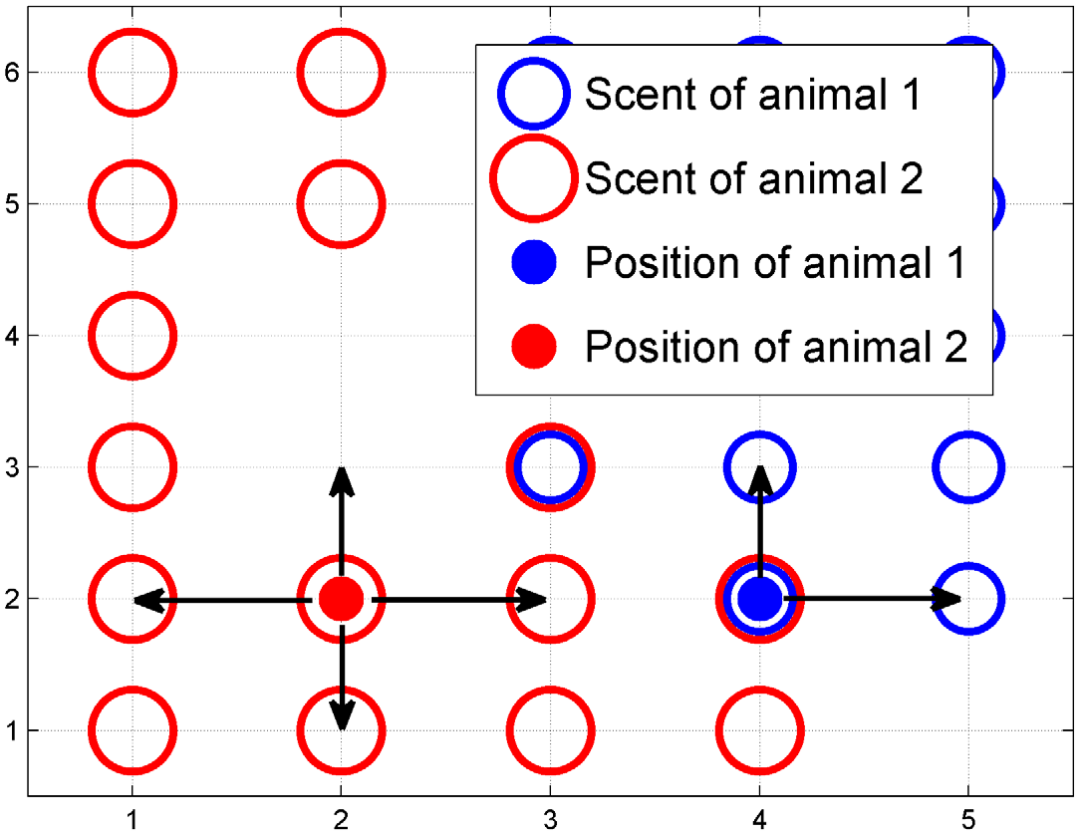
Graphical illustration of the scent-mediated avoidance interaction. This plot shows the possible movement of an animal inside its own territory and when it encounters a foreign scent mark. The figure represents an hypothetical snapshot in time of the position of two animals, the red and blue dots, and their own scent profile, the red and blue open circles, respectively. Wherever red (blue) open circles are present it means that the red (blue) animal has walked over that location in the past 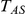 timesteps, where 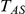 is the period during which scent remains active. The absence of any scent marks at coordinates (5,1) and (2,4) implies that no animal has occupied those coordinates within a time 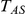. The interaction occurs whenever an animal is occupying a site with a foreign scent as displayed for the blue animal at position (4,2). Since the blue animal has deposited scent at (4,2), this point will eventually become blue territory if the red animal does not re-scent it before the red scent becomes inactive. The subsequent allowed locations where the blue animal can move are those for which no red scent is present, i.e. towards the coordinates (5,2) or (4,3), with the actual movement picked at random from these two possibilities. On the other hand, in the absence of an interaction, an animal such as the red one at coordinates (2,2) can move randomly in any of the four possible directions.

We have used data from the so-called ‘hinterland markers’ [16], [17] which, in our model, deposit scent marks throughout their territory as they move freely over a homogenous terrain as discrete time random walkers on a lattice [18] until they encounter a foreign scent mark, deposited by a neighbouring individual, from which they subsequently retreat in a random direction (see Fig. 1 for a detailed visual illustration of the movement and interaction mechanisms). Occasionally, as a slight modification of the above model, a second version is used that incorporates a variable degree of correlation in the choice of the animal's direction, but only for a short time after the encounter of foreign scent. However unless otherwise stated, we assume the first model throughout the paper.

As the terrain gets covered with scent marks released by the different individuals, each animal is segregated within an area delimited by the locations where the scent marks of neighbours are present, that is, the territory. However, since animals only respond to fresh scent, we consider the scent no longer present after time 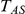 (active scent time). As a consequence the bounded domain where animals are allowed to roam is constantly changing, and the movement of the territories depends on the past locations visited by each animal. A territory is thus a dynamic quantity whose shape and centroid location change continuously.

The rate of movement of a territory depends on the value of the active scent time 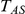; the longer 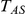 the less the territorial shift (see the Supplementary video S1 and S2 for a visual illustration of territorial movement dynamics for two different values of 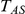). The two extreme situations occur when 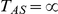 and 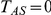, corresponding, respectively, to immobile territories and to territories trivially associated with the instantaneous location of each animal. For intermediate values of 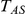, the territories are like deformable elastic objects (see [19] for the elastic disc hypothesis and [3] for its interpretation in terms of territory compression) whose collective dynamics are characterized by the so-called *exclusion process* [20], [21], which prevents the instantaneous occupation of the same location by two or more territories. This exclusion comes about because animals retreat from the locations where they encounter foreign scent, making possible the spatial overlap of different scents only at the boundaries. Territories cannot freely move over the terrain and get hampered, resulting in their movement rates being qualitatively different from that of the animals, the latter being diffusive and the former being subdiffusive [22]. The animal/territory dynamics are thus composed of two time scales, a relatively fast one associated with the diffusive movement of the animals inside their own territory and a slower one associated with the movement of the territories themselves. This latter time scale is controlled by the neighbours' pressure and the time necessary for the resident animal to defend its own borders by refreshing the scent marks. The qualitative difference becomes evident when comparing the variance of the occupation probability, i.e. the mean square displacement (MSD), of an animal and its territory centroid. In 2D these increase respectively as 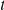 (linear in time as an ordinary diffusive process) and 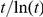 (sublinear in time as a subdiffusive process), the latter due to the exclusion process [20].

**Figure 2:**
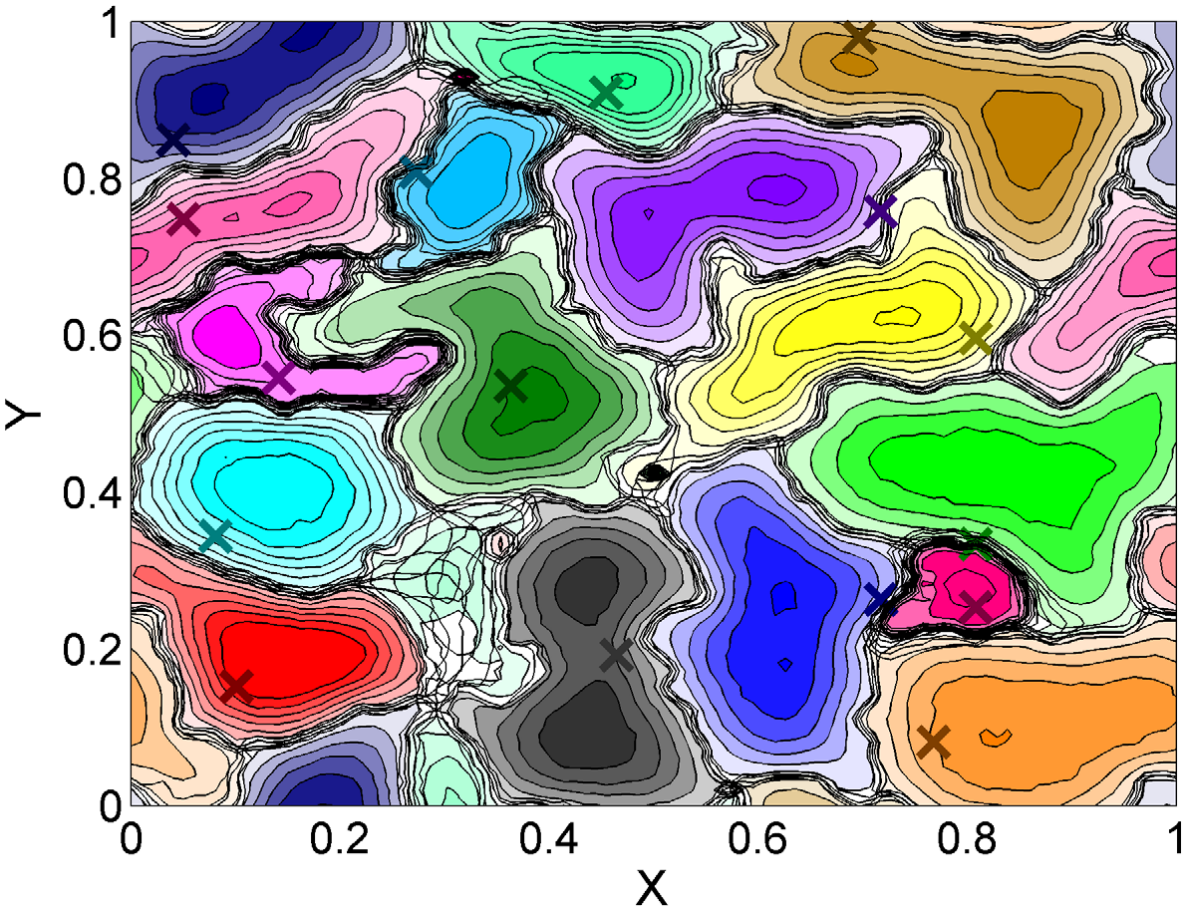
Contour level plot of the utilization distribution. 2D plot of the relative frequency distribution of 16 animals' locations with periodic boundary conditions observed up to time 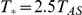 (density is 0.0016 animals per site). The positions 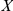 and 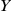 are spatial coordinates normalized to the size of the box. On moving away from foreign scent, the animals perform a correlated random walk with turning angles drawn from a 2-sided exponential distribution with a parameter proportional to 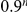, where 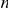 is the number of steps since last encountering foreign scent. The coloured crosses represent the initial animal locations from which their trajectories started to be recorded. This initial condition is obtained from a single run of the simulation up to time 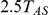, starting from uniformly distributed animals with no initial scent.

An indicator of territorial behaviour is often associated with the size of an animal's territory but for practical convenience territories are often characterized through time-integrated measures which look at the size of an animal's home range [23] and the extent of the home range overlap [24]. This procedure involves tracking the animal's locations through time for a period of observation 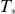 and then selecting a computational method, e.g. the minimum convex polygon (MCP) [25] or the utilization distribution [26], to determine the area delimiting where each animal spends most of the time for its daily activity. In Fig. 2 we show a typical contour level plot of the utilization distribution of a population of 16 individuals obtained from our model. The remarkable feature that becomes apparent in looking at such a plot is that the heterogeneity in space use of the animals emerges dynamically from their interactions without the need to consider any heterogeneity in resource distribution, supporting the idea that territoriality is tightly linked to animal behavioural traits [27] and not just a response to defend potential food and other resources [3]. The heterogeneity in the spatial patterns are in fact only due to the stochastic nature of the interactions as well as the initial animal locations. Features that are commonly observed in field observations [28], such as unequal home range size, boundary areas with small size home ranges (e.g. the pink home range towards the bottom right hand side) squeezed between larger ones, and spatial regions (territorial interstices) which are rarely occupied (e.g. the area between the black, red and dark green ranges), also emerge from our model.

**Figure 3:**
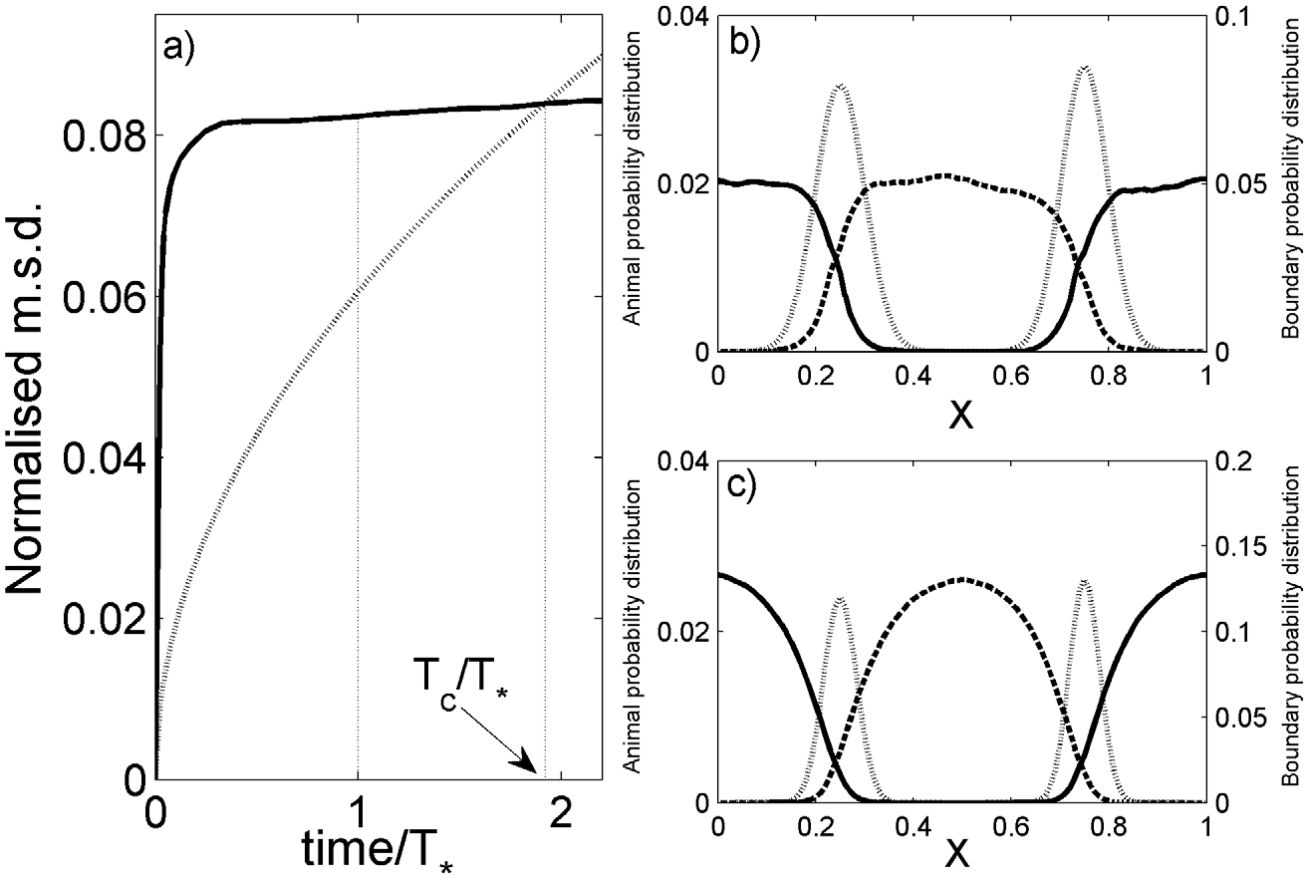
Mean square displacement of the locations of the animals and territorial boundaries. Boundaries are represented by dotted lines, animal 1 by solid lines and animal 2 by dashed lines. In (a) we have plotted the time dependence of the MSD of an animal, 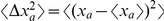 (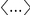 represents an average over the stochastic realizations of multiple trajectories starting with the same initial conditions), and the sum of the left and right boundaries, each 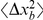, adjusted to correspond to a 90% MCP estimation (see the ‘Relationship between home range size and overlap and mean square displacement’ section of Materials and Methods). Both animals exhibit the same time-dependent MSD so only one is plotted. The choice of the observation time span, from zero up to time 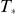 in the figure, determines the degree of territoriality one may infer from the data, the ratio of the adjusted boundary and animal mean square displacements being proportional to the square-ratio of the overlap to the size of a home range (see Materials and Methods). The probability distribution as a function of the spatial position 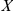, relative to the box size, of the locations of the boundaries and animals at time 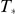 are plotted in (b) and (c), representing the different types of reaction to the encounter of foreign scent marks corresponding to the two versions of our movement model: (b) a random walk movement after retreat and (c) a correlated random walk, where the probability of continuing straight is 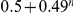, where 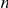 is the number of steps since the animal last encountered foreign scent. These side plots illustrate the role the type of movement performed by the animals may have on the shape of their probability distribution.

To obtain a more quantitative understanding of the relation between the territory and the animal movement rates, we studied in detail a simplified version of our model with two animals in a 1D box with periodic boundary conditions. Since this reduced system also has a time scale disparity between the subdiffusive movement rate of the territories and the diffusive movement rate of the animals, it captures the fundamental characteristics of the animal/territory dynamics in 2D, with the advantage of reducing considerably the computational time of our stochastic simulations. The dynamic nature of the emergence of animal territoriality can be appreciated by plotting in Fig. 3a the variance of the occupation probability, the so-called MSD, of the animal and territorial boundary locations in our model, respectively, 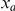 and 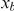. The disparity in movement rates between the boundaries and the animal is evident, with the former being subdiffusive and proportional to 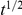. The reduced dimensionality in 1D exclusion processes hinders the movement of the territories even more than in 2D, giving rise to an even slower MSD. The animal MSD, on the other hand, first increases linearly with time and then proportionally to 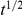 as well, once the increase in the MSD is only due to the random displacement of the territorial boundaries. As the boundaries may roam over the entire space, the sum of the home range overlaps eventually equals the animal's home range at the crossing time 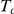, as indicated in Fig. 3a. Although the animals' behaviour is still territorial beyond 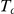, the measurement would suggest that they probably do not possess any exclusive area because the distance from each territorial center is equal to half the width of each animal probability distribution [29]. This dependence on the experimental observation time 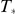 becomes particularly relevant in comparative analyses of territory sizes but it is often overlooked [30]. Meta-analysis of animal territory size should thus be performed by maintaining the same ratio 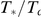 for any species.

**Figure 4:**
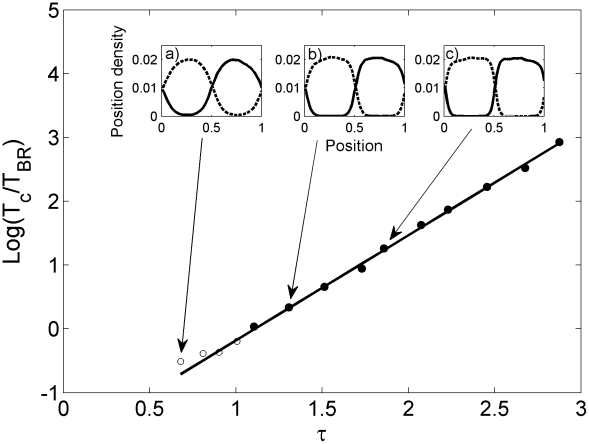
Exclusivity of space use. Cross-over from territories with an area of exclusive use to ones without in terms of 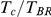 versus 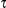. The situation where exclusivity arises is indicated by the closed circles, whereas the absence of exclusivity is represented by the open circles. For a fixed observation time 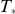, the insets indicate the probability distribution of the two animals as a function of the spatial position relative to the box size. The degree of overlap between territorial neighbours diminishes as 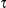 increases, as indicated by inspecting the insets (a), (b) and (c) sequentially. The reaction to the neighbouring scent encountered is the one employed in Fig. 3b and the ratio 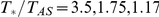 as one moves from inset (a) to (c).

Since a territorial boundary does not change location if an animal has moved over it within a time 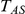 from when it was originally scent marked, the longer it takes for an animal to move back and forth between the foreign scented areas, the more frequently the territorial borders move. As an animal deposits fresh scent at any location it wanders through, the amount of time necessary for this back and forth movement is determined by the temporal extent of two events: traversing from one boundary location to another, and subsequently returning to the starting position. The probability of either of these events occuring is precisely the first-passage probability [31] to reach one boundary location having started at another. We call the sum of the mean of each probability the boundary return time 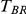, and we define the ratio 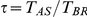 representing the frequency of boundary encounters relative to the frequency of boundary loss. 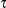 is the fundamental quantity controlling territorial emergence and allows us to compute the movement of the territorial boundaries in terms of the ‘microscopic’ dynamics of the animals. For 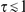 boundaries move so often that 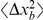 rapidly becomes equal to 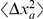 (as shown in Fig. 3a), giving a low crossing time 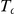. In such a scenario, unless the observation time window 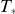 is less than 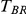, the territories will most likely have no area of exclusive use as shown in the inset (a) of Fig. 4, where the occupation probability at time 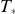 is depicted. For 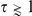, on the other hand, the movement of the territorial boundaries is reduced and areas of exclusive use are likely to emerge (see insets (b) and (c) in Fig. 4).

To verify that meaningful active scent time values can be recovered from movement data, we apply our theory to the red fox (*Vulpes vulpes*) [32] population in Bristol (UK), before the 1994-6 mange epizootic which decimated the fox population [33]. The data we analyze are ideally suited to test our model assumptions since food was widely distributed and available to excess on the study area. We have attempted to use both the 2D as well as the 1D version of our model, the latter one by projecting the experimental observations onto the line connecting the centroids of each animal's home range. Whilst the 2D model is ostensibly more realistic, it does not reflect the fact that foxes tend to snoop around the territory boundaries on each visit. In the 1D model the boundary just consists of two points, so each visit causes half the boundary to be scented. The analyses in the two cases gave results (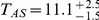 days for 2D, and 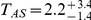 days for 1D), the latter of which agrees well with the time lags of 3–4 days of territorial takeover following the death of all the residents, as observed by one of us (SH) during the mange epizooty in Bristol. We have also examined a variation of our model whereby each animal snoops round a proportion 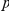 of the territory perimeter on each visit. By increasing 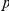, the estimated 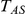 can be reduced. Particulary, a 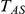 of 3–4 days requires 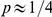. This suggests that foxes are deliberately spending time scenting the boundary, in addition to the movement patterns described in our model.

## Discussion

Mechanistic approaches to territorial behaviour may be limited in scope when mathematical predictions can only be tested at the ‘macroscopic’ population level [3]. Here we have provided a quantifiable ‘microscopic’ testable hypothesis on the mechanism of conspecific avoidance that relates a physio-ecological animal characteristic, the active scent time, to the population distribution of territorial patterns and promises to enrich the emerging field of conservation physiology [34].

To study the role of scent marking in the emergence of territories, one requires the use of modelling techniques that go beyond deterministic reaction-diffusion formalisms [11], since the latter are not viable approximations when the stochastic interaction events are rare and spatially heterogeneous [13]. In territorial formation and maintenance these events are precisely the random encounters of a resident individual with the scent boundaries of a neighbouring conspecific. Moreover, the type of reaction-diffusion model, recently employed to study territorial formation in wolf-packs [12], contains a fundamental constraint: the *a priori* assignment of the location of the focal activity points (e.g. a den or burrow) towards which each animal is attracted [35]. In other words, one of the outcomes of the formation process is predetermined.

By borrowing concepts from non-equilibrium statistical physics, we are able to explain that the different rates of movement between the territories and the animals is the result of geometric constraints coming about because of exclusion processes and the ensuing anomalous sub-diffusive properties of the animal scent profiles. This conceptual framework has allowed us to quantify territorial dynamics in relation to the time an animal needs to move across its own territory refreshing its own scent marks, and the time the chemical signals present in the scent remain active. Although anomalous diffusion is recognized [36] as a useful framework to interpret statistically animal movement data in the context of foraging strategies (see e.g. [37]), our study is the first to show its relevance to animal social interactions.

The necessary level of biological realism that our agent-based simulations introduce is at the expense of mathematical complications since an animal location and its territorial scent profile depend on the history of the other animal trajectories; in other words it is non-Markovian [38] in character. As a consequence each animal perceives an environment which is being modified by the spatio-temporal trajectories of other individuals, making the animal movement dynamics context-dependent [39]. From a single animal perspective, this context-dependence generates a fluctuating heterogeneous environment, which manifests itself in the transient territorial patterns of the individual animals. These findings lend support to the idea that interactions are key to territorial emergence and why no significant effect of resource abundance on territory size has been found in many experimental studies [3].

Although our results are based on considering only individual animals defending a territory, it has a wide applicability since the vast majority of mammals do not form groups, and among these territorial defense is performed by one animal, usually the male, or, in those cases where both sexes play a role, the male generally takes the dominant role. In those cases where groups defend a communal territory but move together (e.g. wolves), it makes little difference whether one or all of the animals scent mark or play a role in territorial defense, since their movement behaviour will be little different from the solitary animal represented in our model.

We have tested the applicability of our model by verifying that a meaningful active scent time can be obtained from urban red fox data, that is data from a territorial species moving within an environment where food is widely and abundantly available. This result and the simplicity of our assumptions in the movement of the animals implies that our study has produced a null model of animal interactions onto which one can add nutritional effects [40] and test optimality questions [41] in cooperative search strategies [39], [42].

Besides the relevance to territorial formation, the results of our model represent a benchmark to test ideas related to the role of scent in animal communication, and in particular in the context of foxes and possibly other carnivores. Fox scent marks provide a great deal of information about the fox that left the message, because chemical analyses of the volatile compounds present in fox scent marks can identify sex, season, relatedness, health and possibly social status of an animal [43]; whilst it is currently unknown whether foxes can also receive all of these messages, it seems highly probable that they do. This would then explain why foxes snoop: they can learn all they need to about their neighbours, both presence and more detailed information, without ever needing to meet. In this respect our model provides a useful tool to help design field experiment to study behavioural response of resident animals to alien scent marks from animals of known age, sex and social status, both in the snoop zone and further into the territory.

It is relevant to note that the importance of the frequency of animal encounters has been considered in studies of the allometric scaling of the exclusivity of space use [44], by using an analogy between animal encounter rate to the interaction frequency of physical particles in an ideal gas [45]. In that context our results provide a spatially explicit ‘microscopic’ interpretation of that study [44].

In summary, with a systems biology type of approach we have been able to show that scent marks in territorial animals serve as a short-term cue, illustrating why territorial mammals regularly renew and refresh their scent marks. Our field observations on foxes showed that, when territories became vacant, they were rapidly taken over by neighbours and our model demonstrates this very neatly. We have also shown the practical steps required to extract active scent time from 2D animal fixes.

## Materials and Methods

### The stochastic simulations

The positions of the interacting random walkers on a lattice are updated at every time-step following the rules depicted in Fig. 1. In order to measure the walker probability distibutions over time, initial conditions in the stochastic simulations are expressed in terms of the animal locations and the scent spatial profiles, i.e. each lattice site defines the time the scent is still available before it becomes inactive. A biologically relevant initial scent profile is a curve with minimum values at the scent boundaries and a maximum at the lattice site where the animal is initially present. Since territories may move only when the scent profile at the boundary of at least one of two neighbouring animals is equal to zero, we have used an initial scent profile with such a feature. The shape of the curve, which interpolates between zero at the boundaries and the maximum corresponding to the animal's position, is obtained by averaging over stochastic simulations that are run starting with a spatially uniform distribution of animals and with all scent profiles equal to zero. The moment when this average is computed is at a time 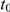 (see table 1) longer than 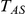, corresponding to the situation when the boundary MSD has reached its asymptotic regime.

**Table 1:** Notation glossary.

Symbol	Explanation	Input/Output
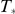	Time-window over which data on animal positions are gathered.	Input
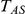	Active scent time: the amount of time that the scent of one animal is considered to be ‘fresh’ by other animals.	Input
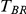	Boundary-return time: the average time for an animal to visit every point on the boundary and return to the point it set out from.	Output
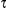	The quotient 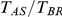 representing the frequency of boundary encounters relative to the frequency of boundary loss.	(see 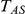 , 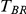 )
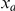	The position of an animal in a simulation.	Output
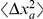	The MSD 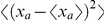 of an animal, averaged across 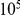 simulated trajectories and starting from the same initial conditions.	Output
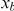	The position of a territorial boundary location.	Output
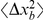	The MSD of a territory boundary. In 1D, this is simply the MSD 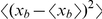 of either of the boundary points 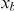 , averaged over 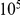 simulations. In 2D, the main contributor to 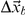 is the displacement of the territory centroid, 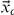 . However, we also added the displacement of the territory radius 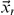 , defined to be the mean distance from the centroid to the boundary points at any instant in time. More precisely 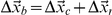 , where 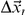 is the average over stochastic realizations along an arbitrarily chosen reference direction (north-east in our choice).	Output
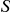	90% MCP home range size.	Output
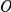	90% MCP home range overlap: i.e. the 90% MCP of the boundary position distribution.	Output
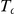	The average time span beyond which we are going to observe areas of exclusive space-use, i.e. the time when 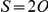 .	Output
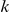	Generalised diffusion constant of the territory boundaries.	Output
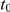	Time at which 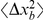 reaches its asymptotic limit (i.e. is proportional to 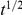 in 1D and 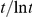 in 2D).	Output

Glossary of the various symbols used throughout the text, a brief explanation of each and whether the quantity is measured from the model (output) or a parameter of the model (input).

In order to measure the boundary position distributions in both the 1D and 2D models, we examined the asymptotic regime, where 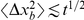 in 1D or 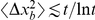 in 2D, so that the movement is independent of initial conditions.

For Figs. 3 and 4 the number of simulations carried out to plot the occupation probabilities and the boundary and animal MSD is equal to 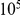. Fig. 2 on the other hand is generated with only 100 simulations since utilization distributions are time-integrated quantities [26] and require less averaging to obtain smooth contour level plots.

### Relation between home range size and overlap and mean square displacement

As territorial interactions are dynamic, and often rely on scent marking, it is hard to detect subtle changes in territorial boundaries using conventional field techniques. So size of animal home ranges is used as a surrogate for territory size. A by-product of the home range estimation for neighbouring individuals is the determination of the area of home range overlap. It is thus of interest to know how to relate the MSD, as shown in Fig. 3, with home range size 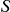 and home range overlap 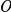. Since boundary movement is the result of an exclusion process, the boundary location distribution function over an extended period is Gaussian [46], so that the boundary MSD is 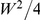, where 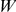 is the width of the boundary location distribution at 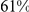 of the distribution's maximum height. For the animal, on the other hand, it is necessary to relate the MSD to the area obtained from the extreme statistics associated with the determination of the MCP [47], [48]. Since the animal and boundary position distributions both have infinite tails, there is an unbounded increase of the 100% MCP as the number of samples from either distribution increases [11]. Therefore we have selected an A% MCP with 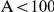 whose width saturates with time. This choice is related to the shape of the probability distribution and we found that a flat-topped curve with exponential tails well approximates the shape of the animal probability distribution for time values relevant in our simulations. We noticed that by employing A = 90% we can relate the 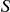 value obtained from the MCP calculation with the MSD through the expression 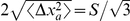 within an error of 5%. From normal distribution tables we know that 90% MCP of the boundary distribution, in other words the 90% overlap 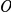, is a factor of 1.645 larger than 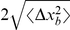 for 1D from which it follows that 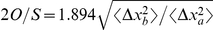. In Fig. 3 the MSD of the animal has been plotted as is, whereas one of the boundaries has been multiplied by 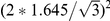 to ensure that the two curves cross where 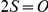. For 2D, the value corresponding to 1.645 is 2.146. This is used in the data-fitting below.

### Analysis of the red fox *Vulpes vulpes* radiotelemetry data

We used data from a long-term study of the red fox population in the Bristol urban area with a spatial resolution of 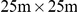, larger than the spatial heterogeneity perceived by the animals. We analyzed radio telemetry data from adult foxes recorded during the springs of 1978 and 1991 and the summer of 1990. Throughout each season, three foxes from different groups were radio-tracked for between 5 and 11 nights, with positional fixes taken every 5 minutes. We employed both the results in 1D and 2D in estimating an active scent time 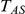 from the radio telemetry data. For the 1D model we projected the animal locations onto a line through their home range centres, calculated by taking the centroid of all position fixes. From the first 25 minutes after a fox begins to move, the diffusion constant (see e.g. [49]) of the animals was estimated to be 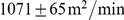, giving a time-step in the model corresponding to 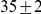 s. Since the red fox is active for just 8 hours in any 24 hour period [50], each 90 day data-gathering time-window corresponds to 720 hours, or an observation time 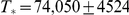 time-steps. Table 2 shows the number of fixes used and the period when data were collected as well as the animal's gender. Although the dominant male is the primary territorial defender, since other adults in the group will have roughly the same home ranges as the dominant male, we have used data from both male and female foxes. However, all the data are from adult foxes since cubs and sub-adults tend to have smaller home ranges [51].

**Table 2:** Data collected on neighbouring foxes.

Fox	Season	Number of radio locations	Gender	Fox density
A	Spring 1978	410	F	0.0257 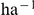
B	Spring 1978	124	M	0.0257 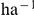
C	Spring 1978	433	F	0.0257 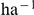
D	Summer 1990	488	M	0.0324 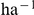
E	Summer 1990	673	M	0.0324 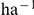
F	Summer 1990	267	M	0.0324 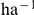
E	Spring 1991	873	M	0.0442 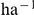
F	Spring 1991	582	M	0.0442 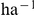
G	Spring 1991	932	F	0.0442 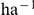

Number of radio locations, season of observation, gender and number of individuals per hectare for the foxes with overlapping home ranges observed between 1978 and 1991.

From extensive simulations of our 1D model we determined that the quantitative dependence of the generalized diffusion coefficient 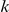 in the boundary MSD 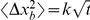 falls onto a universal curve when plotted versus 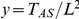 where 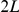 is the size of the periodic box. The fitting line is given by 
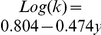
 with 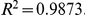 To understand this universal curve we analysed the dynamics of two interacting animals in two extreme situations: 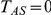 and 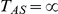. For the case 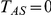, we constructed with the help of diffusion graph transform [52] a discrete Master equation for the relative distance of two random walkers roaming within a lattice of 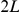 sites with periodic boundary conditions. The two walkers move freely except when they meet, after which they move away from each other. By solving this master equation, we computed the mean first passage time for two walkers, starting in the same square, with walker 1 having just moved left, to meet again with walker 1 having just moved right. This quantity is half the mean boundary return time 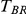 (for the case 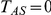) that we have defined in the text. It turns out that 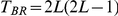. For the other extreme case 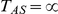, the first-passage time is governed purely by the sizes of the two territories that initially form, since thereafter they do not move. For a random walk restricted to move on a line segment, the mean first-passage time to go from one (reflecting) edge to the other (absorbing) edge is the square of the length of the segment [31]. Assuming that one of the territories is of size 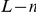 and the other of size 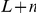, the average 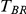 is equal to 
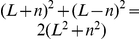
. Let 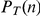 be the probability that the initial territories are of sizes 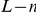 and 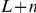. Then 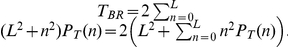 Using numerical simulations for various 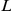, we find 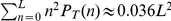 so 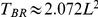. For intermediate values of 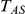 and sufficiently large values of the box size 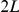, we can expect an 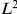 dependence of 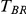 with a coefficient of proportionality interpolating between 4 and 2.072, explaining the universal fitting curve as described above. This dependence is also what one would expect in 2D, where first-passage times in a bounded domain are proportional to the size of the domain itself, to a first order approximation [53]. By projecting position fixes onto the line between adjacent home range centres, we calculated the 90% MCP home range width to be 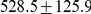 m and the overlap-to-size ratio 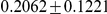. This corresponds to a model density of 1 animal per 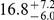 lattice sites and an overlap of 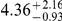 sites. Since the boundary displacement is Gaussian, we can use the 90% home range overlap, which is 
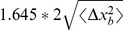
, making the boundary MSD 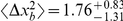. From the theoretical values of the diffusing boundary MSD 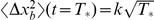, we recover a value of 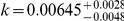. From the universal curve described above and the experimental population density, we relate 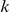 to 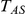, giving 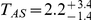 days. The procedure in 2D is similar, although we have not studied in detail if there exists a universal curve of the dependence of 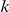 as a function of the box size and 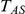. We considered the average population density from the data, which was 1 male dominant fox per 29.3 hectares, and ran the simulation for various 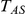. In each case, we considered the asymptotic regime of the boundary MSD and used this to calculate 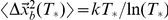 for 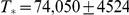 time-steps. The resulting empirical relationship obtained from the stochastic simulation between 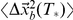 and 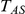 gives us 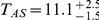 days.

## Supporting Information

Video S1Territorial dynamics with small active scent time. Movie of the territorial dynamics of 25 animals with an active scent time 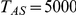 time steps in a box of 100×100 sites with periodic boundary conditions. The initial movie frame is recorded after a small transient obtained from an initial condition with the animals periodically placed on the lattice and without any scent profile. The snapshots of the simulations are taken every 10,000 time steps.(GIF)

Video S2Territorial dynamics with large active scent time. Movie of the territorial dynamics of 25 animals with an active scent time 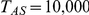 time steps in a box of 100×100 sites with periodic boundary conditions. The initial movie frame is recorded after a small transient obtained from an initial condition with the animals periodically placed on the lattice and without any scent profile. The snapshots of the simulations are taken every 10,000 time steps.(GIF)
