# Supplementary figures and images for: Animal Interactions and the Emergence of Territoriality
Source: PLoS Comput Biol. 2011 Mar 10;7(3):e1002008. doi: 10.1371/journal.pcbi.1002008 (PMC3053310; doi:10.1371/journal.pcbi.1002008)

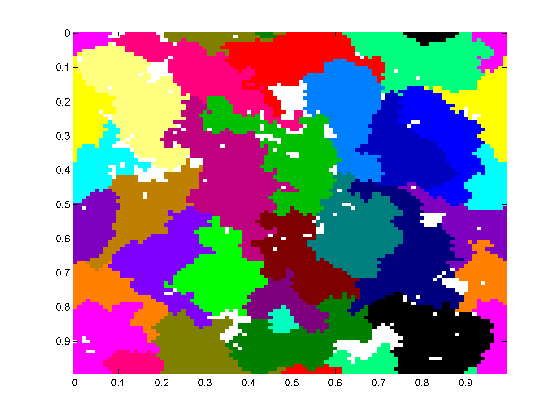

Supplement: Video S1 — Territorial dynamics with small active scent time. Movie of the territorial dynamics of 25 animals with an active scent time time steps in a box of 100×100 sites with periodic boundary conditions. The initial movie frame is recorded after a small transient obtained from an initial condition with the animals periodically placed on the lattice and without any scent profile. The snapshots of the simulations are taken every 10,000 time steps. (GIF) [file pcbi.1002008.s001.gif]

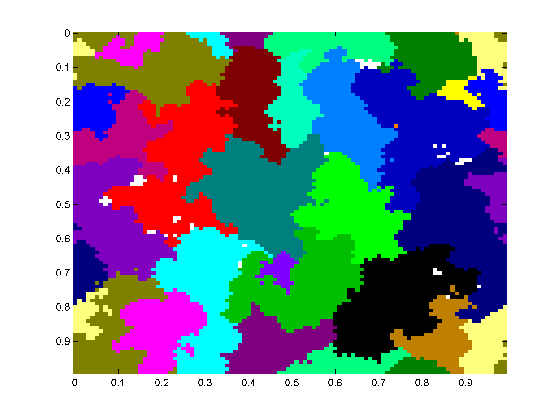

Supplement: Video S2 — Territorial dynamics with large active scent time. Movie of the territorial dynamics of 25 animals with an active scent time time steps in a box of 100×100 sites with periodic boundary conditions. The initial movie frame is recorded after a small transient obtained from an initial condition with the animals periodically placed on the lattice and without any scent profile. The snapshots of the simulations are taken every 10,000 time steps. (GIF) [file pcbi.1002008.s002.gif]
